# Well-preserved Devonian galeaspid indicates swimming performance of jawless fishes

**DOI:** 10.1093/nsr/nwad092

**Published:** 2023-04-12

**Authors:** Philippe Janvier

**Affiliations:** Museum National d’Histoire Naturelle, and Sorbonne Universtité, France

A paper by Gai *et al.* [1] provides the first detailed description of the tail in an exceptionally well-preserved galeaspid from China, and the authors provide some interesting insights into the mode of life and swimming speed in this group of stem gnathostomes. Galeaspids are a very peculiar group of vertebrates. Like osteostracans, which are currently regarded as a sister group to jawed vertebrates, their gills are enclosed in depressions, or gill fossae, of the ventral surface of the braincase and open ventrally to the exterior by minute pores. In some galeaspids there may be ∼50 such gill fossae on each side; that is, the largest number of branchial units ever recorded in vertebrates, along with euphaneropids. The environment and overall aspect of most galeaspids suggest that they were benthic fishes, and that the flux of respiratory water entered the gill apparatus through the median dorsal opening (in fact, a naso-hypophysial duct), and was expelled postero-ventrally via the gill pores after having oxygenated the numerous gills. This peculiar branchial apparatus certainly implies a water-pumping device, comparable or homologous to the velum of extant jawless vertebrates (cyclostomes). A relatively rapid swimming speed may have increased the performance of this mode of gill ventilation.

I am not entirely convinced by the sophisticated calculations provided in this paper, but I entirely agree that the long-unknown caudal fin of galeaspids was basically hypocercal, with a long ventral lobe, like in heterostracans, contrary to the epicercal tail of osteotracans and gnathostomes. The transition from a hypocercal to an epicercal tail, probably by Ordovician times, certainly was a biologically and ecologically important revolution in vertebrate history, even if the thrust produced by the two tail types may have been almost the same, as demonstrated by experiments on gnathostomes. Yet the pitch of the body became different, and probably more efficient when it came to feeding on nektonic or floating organisms and food particles. It would be better to rely on factual data based on extant fish proxies, such as benthic osteichthyans or chondrichthyans.

Nevertheless, it was worth trying to estimate the swimming speed of galeaspids using quantified parameters.
